# Nutrients and Their Role in Autophagy Regulation

**DOI:** 10.1021/acs.biochem.6c00482

**Published:** 2026-10-14

**Authors:** Niall Wilson, Gamze Kocak, Sovan Sarkar, Yoana Rabanal Ruiz, Viktor I. Korolchuk

**Affiliations:** † Biosciences Institute, Faculty of Medical Sciences, 5994 Newcastle University, Newcastle upon Tyne NE4 5PL, United Kingdom; ‡ Department of Cancer and Genomic Sciences, School of Medical Sciences, College of Medicine and Health, 1724 University of Birmingham, Edgbaston, Birmingham BT15 2TT, United Kingdom; § Department of Medical Sciences, Faculty of Medicine of Ciudad Real, Molecular Studies Applied to Health Sciences Group (EMAS), UCLM, Camino de Moledores s/n, 13071 Ciudad Real, Spain; ∥ Department of Clinical Chemistry, Faculty of Allied Health Sciences, Chulalongkorn University, Bangkok 10330, Thailand

**Keywords:** autophagy, cellular homeostasis, endoplasmic reticulum (ER), metabolism, phagophore assembly site (PAS)

## Abstract

Autophagy is a fundamental catabolic pathway that maintains cellular homeostasis through the degradation and recycling of intracellular components in response to nutrient limitation and metabolic stress. Increasing evidence indicates that autophagy is tightly regulated by cellular metabolism, with nutrients and metabolites acting not only as energy substrates and biosynthetic intermediates, but also as signaling molecules that directly modulate the autophagic machinery. In this review, we discuss how different metabolites regulate autophagy through interconnected nutrient- and energy-sensing pathways. We further examine how these metabolic signals coordinate distinct stages of autophagosome biogenesis and autophagic flux under conditions of nutrient deprivation or energy stress. Collectively, metabolite-autophagy crosstalk emerges as a central mechanism that enables cells to balance metabolic demand with intracellular resource availability, while its dysregulation contributes to metabolic imbalance and age-associated pathology.

## Graphical abstract

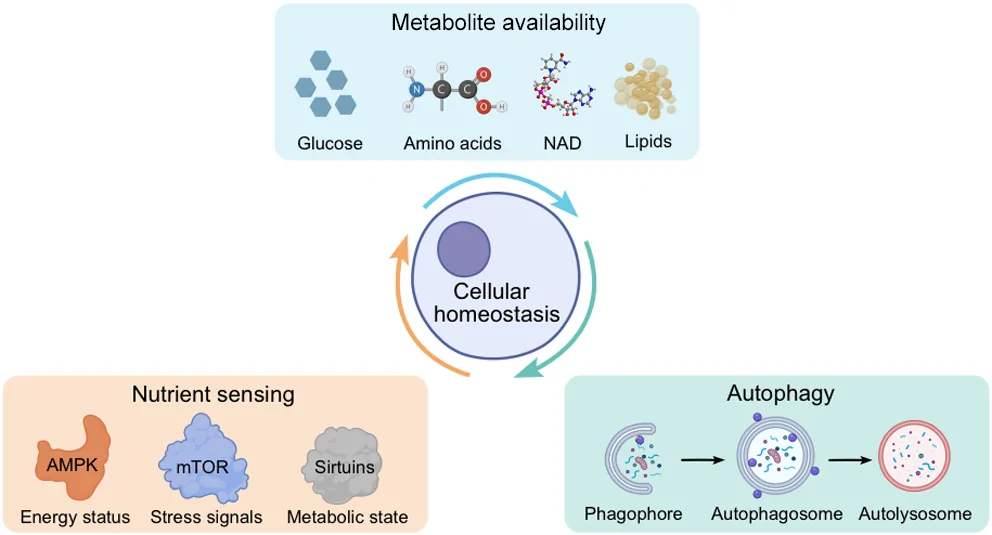

## Autophagy as a Metabolic Pathway

Autophagy is a highly conserved lysosomal degradation pathway responsible for the turnover and recycling of cytoplasmic material.^1^ Among the well-defined autophagy pathways, macroautophagy (hereafter referred to as autophagy) is the primary route for the bulk turnover of proteins, organelles and metabolites.^2^ Although traditionally viewed primarily as a survival and quality control mechanism induced by nutrient deficiency, autophagy also operates constitutively to maintain basal metabolic homeostasis.^3^ Therefore, in addition to acting as an emergency response, autophagy continually balances metabolite demand with intracellular resource availability.

This constant flux through the autophagy pathway has important consequences for cellular metabolism. By degrading macromolecules and organelles, autophagy contributes to the maintenance of diverse metabolite pools, including amino acids, lipids, sugars, nucleotides and redox cofactors.^4–6^ Accordingly, autophagy acts as a metabolic buffering system, providing a flexible source of substrates that can be redirected toward energy production and stress adaptation across shifting nutritional states. Consistent with this role, autophagy is tightly integrated with nutrient- and energy-sensing networks, including kinase signaling pathways, redox-sensitive mechanisms and metabolite-dependent post-translational modifications, that collectively adjust autophagic flux according to cellular demand.^7^

Within this framework, metabolites act not only as products of autophagic degradation, but also as active regulators of the pathway itself, shaping both the activity and selectivity of autophagic degradation. Following a brief overview of the molecular machinery underlying autophagy, we discuss how various nutrients and metabolites regulate this system. By integrating these perspectives, we argue that autophagy represents a dynamically regulated and metabolically integrated pathway, continuously tuned by metabolite availability to preserve cellular function.

## Molecular Machinery of Autophagy

Autophagy induction requires more than 35 autophagy-related genes (ATG) that operate in a tightly coordinated fashion and involves the sequential activation of protein complexes that drive the formation of autophagosome precursors (phagophores). These membrane precursors recruit the molecular machinery required for membrane expansion, cargo recognition and engulfment into double-membraned structures formed *de novo* called autophagosomes. The process culminates in the fusion of autophagosomes with lysosomes, where the enclosed cargo is degraded into basic metabolites that can subsequently be recycled for energy production or the synthesis of new cellular components (Figure 1).

**Figure 1:**
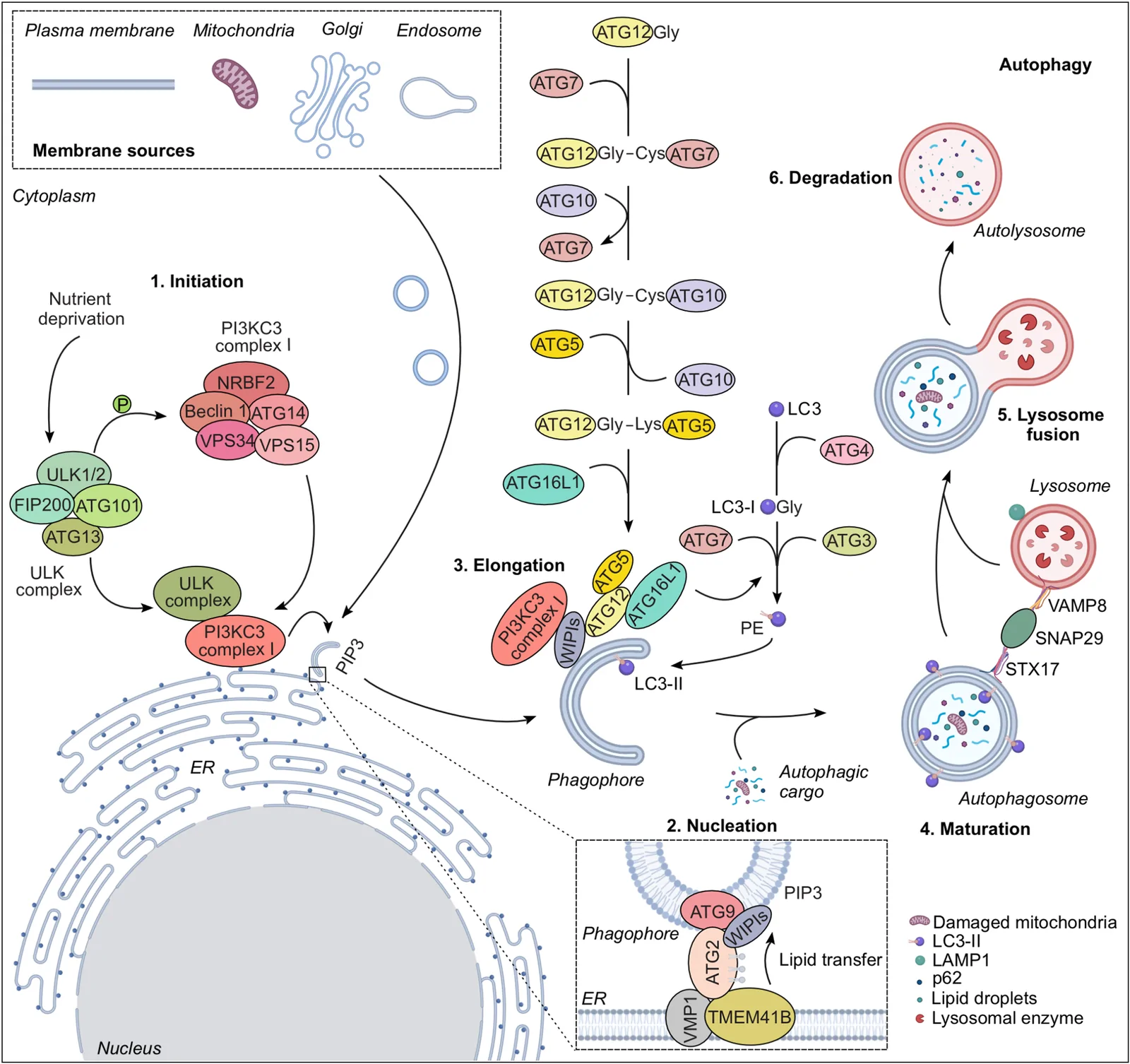
Molecular machinery of autophagy pathway. Schematic overview of the molecular mechanisms regulating autophagy. Autophagy is initiated in response to nutrient deprivation and cellular stress through activation of the Unc-51-like (ULK) complex, composed of ULK1/2, autophagy-related protein 13 (ATG13), RB1-inducible coiled-coil protein 1 (FIP200), and ATG101. Assembly and activation of the ULK complex promote recruitment of the class III phosphatidylinositol-3-kinase complex I (PI3KC3-C1), consisting of vacuolar protein sorting 34 (VPS34), VPS15, Beclin 1, ATG14 and NRBF2, leading to phosphatidylinositol-3-phosphate (PI3P) production at endoplasmic reticulum (ER)-associated membrane sites. During phagophore nucleation, PI3P recruits WD repeat domain phosphoinositide-interacting proteins (WIPIs) to specialized ER subdomains known as omegasomes. Membrane sources from the ER, plasma membrane, mitochondria, Golgi, and endosomes contribute to phagophore formation and expansion by supplying membrane material, including lipids delivered through ATG9A-containing vesicles. Lipid transfer from the ER to the expanding phagophore is mediated by ATG2, while phospholipid scrambling and membrane expansion are facilitated by the ATG9A together with the ER-resident proteins vacuole membrane protein 1 (VMP1) and transmembrane protein 41B (TMEM41B). Elongation is mediated by two ubiquitin-like conjugation systems: the ATG12-ATG5-ATG16L1 complex and LC3 lipidation. ATG12 is sequentially conjugated through ATG7 and ATG10 to ATG5 and associates with ATG16L1, whereas microtubule-associated protein chain 3 (LC3) is processed by ATG4 and conjugated to phosphatidylethanolamine (PE) via ATG7 and ATG3, generating membrane-associated LC3-II. The expanding phagophore sequesters cytoplasmic cargo, including damaged organelles and protein aggregates, to form a double-membrane autophagosome. During maturation, autophagosomes acquire fusion competence and subsequently fuse with lysosome through soluble N-ethylmaleimide-sensitive factor activating protein receptor (SNARE) proteins, including syntaxin 17 (STX17), synaptosomal-associated protein 29 (SNAP29), and vesicle-associated membrane protein 8 (VAMP8). The resulting autolysosome enables degradation of the engulfed cargo by lysosomal hydrolases, thereby recycling cellular components and maintaining cellular homeostasis.

### Autophagosome Biogenesis

The autophagy initiation complex consists of 4 components: Unc-51-like autophagy-activating kinases 1/2 (ULK 1/2), the RB1-inducible coiled-coil protein 1 (FIP200; also known as RBCC1) and the HORMA (Hop/Rev7/Mad2) domain-containing proteins Atg13 and Atg101.^8,9^ The assembly and activation of this complex is driven by ULK1 autophosphorylation and additional kinase-mediated modifications leading to the formation of punctate structures near the endoplasmic reticulum (ER) membrane that triggers the nucleation of the phagophore. Phagophore formation also requires the recruitment of the second autophagy core complex, the Class III PI3P kinase (PI3K) Vacuolar Protein Sorting 34 (VPS34) complex (PI3KC3-C1) to the phagophore assembly site (PAS). The PI3KC3-C1 complex, constituted by the class III PI3K VPS34, VPS15, Beclin1 ^10^ and ATG14, triggers site-specific production of phosphatidylinositol-3-phosphate (PI3P) at a specialized ER structure known as the omegasome,^11,12^ a platform from which autophagosome emerges. NRBF2, which binds with high affinity to VPS15, is also frequently considered a fifth component of the complex.^13–15^ NRBF2 forms homodimers that are required for the integrity of PI3KC3-C1,^16^ although its precise role in regulating complex activity remains incompletely understood.^15,17,18^

These complexes engage in a bidirectional interplay that coordinates autophagy initiation at the PAS. Their physical interaction, mediated by the FIP200 arm region and VPS15 tail domains,^19^ facilitates PI3KC3-C1 recruitment to the PAS. In turn, ULK1 phosphorylates all four subunits of the PI3KC3-C1 complex,^20–22^ enhancing its catalytic activity and promoting PI3P production. Additionally, PI3KC3-C1 induces conformational changes in ULK1 that favor its dimerization and autophosphorylation, thereby supporting full ULK1 activation.^19^ Together, these reciprocal interactions promote efficient autophagosome biogenesis through coordinated membrane-associated activation of both complexes.^19^

### PI3P-Mediated Expansion of the Phagophore

PI3P generated by PI3KC3-C1 drives the expansion of the phagophore membrane through the recruitment of WD repeat proteins interacting with phosphoinositides (WIPIs), such as WIPI1, WIPI2, WIPI3, and WIPI4, which subsequently promote the binding of the lipid transfer protein ATG2.^23,24^ Concomitantly, ATG9-containing vesicles originating from the secretory pathway are recruited to the PAS.^25^ Both ATG2 and ATG9 cooperate to support phagophore elongation and autophagosomal membrane expansion.^26^ ATG2 acts both as a tether between ER and phagophore membranes and a lipid transfer protein, mediating phospholipid transport from the outer leaflet of the ER membrane to the outer leaflet of the growing phagophore.^27^ In parallel, ATG9 forms a homotrimeric complex embedded within the phagophore membrane that functions as a scramblase, facilitating phospholipid translocation between the outer and inner membrane leaflets, thereby coordinating with ATG2-mediated lipid transfer.^26,28^

In addition, the expansion of the autophagosome requires the binding of PI3KC3-C1, via WIPI2,^29^ to two ubiquitin-like conjugation systems which catalyze the covalent attachment of ATG12 to ATG5 and ATG16-like 1 (ATG16L1) and that of phosphatidylethanolamine (PE) to ATG8/LC3.^30,31^ ATG12 and LC3 function as ubiquitin-like proteins within their respective conjugation systems, and both require the E1-like enzyme ATG7 for activation. ATG8 proteins (LC3/GABARAP) are subsequently transferred to the E2-like enzyme ATG3 which presents an amphipathic helix that associates with high curvature membranes or those containing conical lipids, such as PE.^32^ The efficient lipidation and recruitment of LC3/GABARAP in vivo requires the E3-like activity of the ATG12-ATG5 conjugate^33^ which associates with ATG16L1 to form a multimeric ATG12-ATG5-ATG16L1 complex. This complex is recruited to the PAS through interaction of ATG16L1 with WIPI2 ^34^ and serves as the E3-like enzyme for the ubiquitin-like conjugation of LC3 family members to the PE.^35,36^ This conversion generates a lipidated, membrane-bound form (e.g., LC3-II) from a cytosolic precursor (e.g., LC3-I). Conjugation of ATG8 proteins to PE can cause membrane perturbations that trigger efficient phagophore expansion and may also contribute to membrane closure.^37,38^ This process is proposed to occur at ER exit sites (ERES).^39^ Nevertheless, additional membrane sources including the Golgi apparatus, ER-Golgi intermediate compartment (ERGIC), ER-mitochondria contact sites (MAM), recycling endosomes or plasma membrane have also been reported to contribute to autophagosome formation.^40–42^

Depending on the nature of the cargo incorporated into these membranes, autophagy can be broadly classified into selective and nonselective pathways. Nonselective autophagy involves the bulk sequestration of cytoplasmic material, including proteins and organelles. In contrast, selective autophagy specifically targets defined cellular components such as protein aggregates and damaged organelles, including mitochondria, peroxisomes, lysosomes, endoplasmic reticulum, and even parts of the nucleus.^43^ This selectivity is mediated by autophagy receptors, such as sequestosome-1 (p62/SQSTM1), NDP52/CALCOCO2 or optineurin (OPTN), which link cargo destined for degradation to the autophagosomal membrane. The presence of LC3-interacting regions (LIRs) or GABARAP-interacting motifs (GIMs) in these receptors enables direct binding to ATG8-family proteins, ensuring selective and efficient recruitment of substrates into autophagosomes.^44^

### Autophagosome-Lysosome Fusion

Following cargo sequestration, closure of the phagophore into a double-membrane autophagosome requires membrane scission mediated by the ESCRT machinery.^45–47^ Mature autophagosomes subsequently fuse with lysosomes to form autolysosomes, where cargo degradation occurs. Autophagosome-lysosome fusion is coordinated by ATG8-family proteins together with SNARE complexes, including STX17-SNAP29-VAMP7/8 and YKT6-SNAP29-STX7, while additional tethering factors such as HOPS, EPG5, PLEKHM1, BRUCE, and GRASP55 cooperate with Rab7 and LAMP2 to facilitate membrane fusion.^48–53^ Lysosomes constitute the terminal degradative compartment of autophagy, where more than 60 acidic hydrolases (proteases, lipases and nucleotidases) digest autophagic cargo into reusable metabolites that are exported from the lysosomal lumen to the cytosol through a variety of lysosomal membrane transporters. This process relies on proper lysosomal acidification, which is maintained by the vacuolar H^+^-ATPase (v-ATPase). Notably, this v-ATPase has also been shown to induce a selective autophagic response, further highlighting the connection between lysosomal function and autophagy regulation.^54,55^

## Nutrient and Metabolite Sensing in Autophagy Regulation

Autophagy responds to nutrient starvation to varying degrees, depending on the metabolites and the extent to which they are deprived. Indeed, nutrient availability regulates the localization and activity of core autophagy complexes, including ULK1, VPS34, and ATG12-ATG5-ATG16L1 ^56^ and reflects the overall energetic and biosynthetic state in an integrated manner through nutrient-sensitive kinases that transmit these signals to downstream autophagy complexes and ATG proteins to control autophagy. Consequently, nutrient-dependent regulation of autophagy is mediated through multiple interconnected signaling axes that translate metabolic inputs into autophagic output. Among the central regulators of these nutrient-sensing pathways are AMP-activated protein kinase (AMPK) and the mechanistic (or mammalian) target of rapamycin (mTOR). These kinases act as master regulators of autophagy initiation.^57^ The diverse mechanisms by which nutrients regulate autophagy and cellular signaling are summarized in Figure 2.

**Figure 2:**
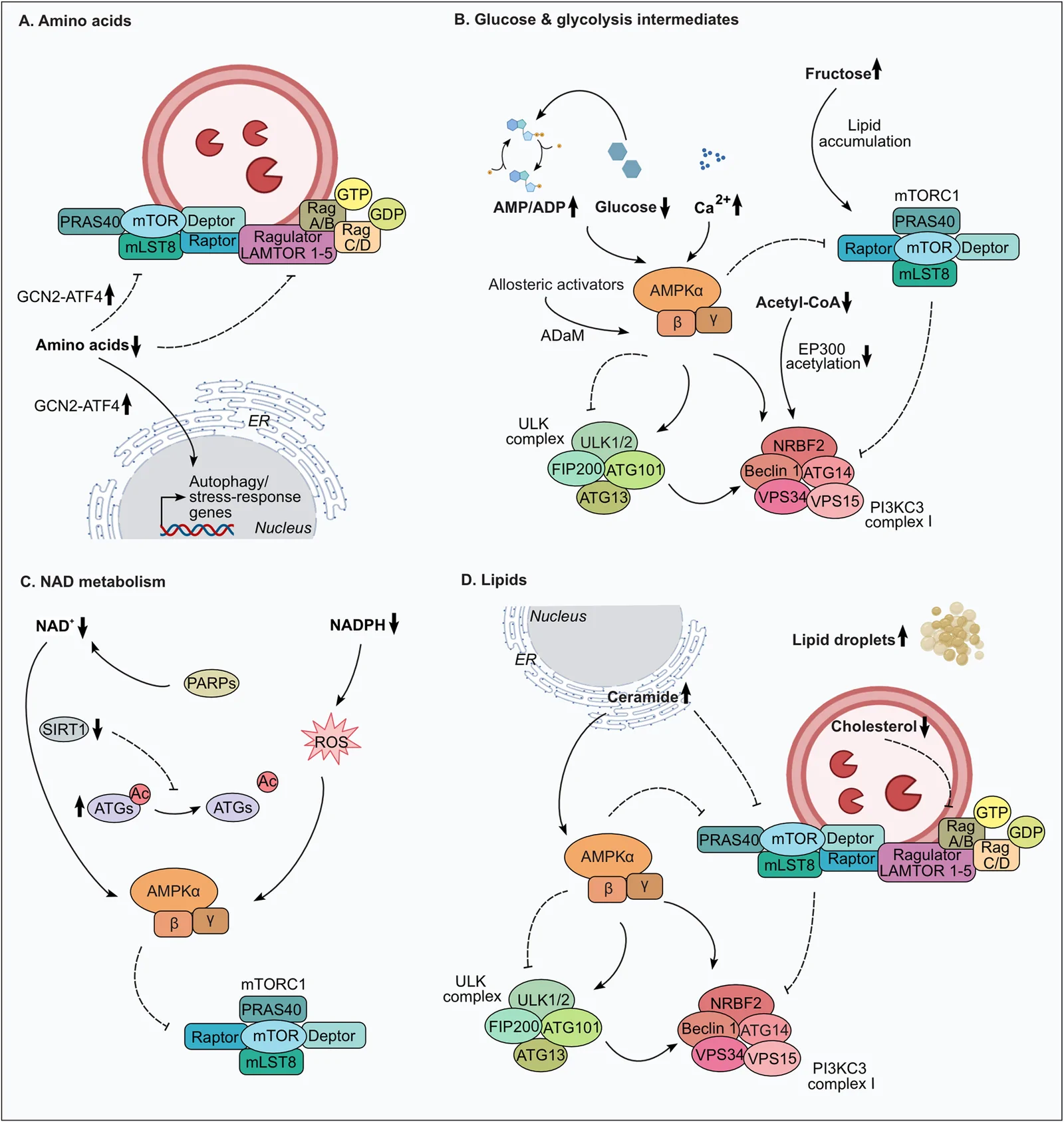
Metabolite-dependent nutrient sensing pathways regulating autophagy. Schematic illustration of selected nutrient and metabolite deprivation signals converge on key nutrient-sensing pathways to modulate autophagy. (A) Amino acids. Amino acid deprivation suppresses mechanistic (or mammalian) target of rapamycin (mTORC1) activity through lysosomal nutrient sensing involving Ragulator (LAMTOR1–5) and Rag GTPases (RagA/A-RagC/D), while simultaneously activating the general control nonderepressible 2- activating transcription factor 4 (GCN2-ATF4) pathway, which additionally promotes autophagy- and stress-response gene expression. (B) Glucose and glycolytic intermediates. Reduced glucose availability increases AMP/ADP ratios and intracellular calcium (Ca^2+^), promoting AMP-activated protein kinase (AMPK) activity and downstream signaling to the unc-51-like kinase (ULK) and class III phosphatidylinositol 3-kinase (PI3KC3) complex 1. Decreased cytosolic acetyl-coenzyme A (acetyl-CoA) availability decreases EP300-mediated acetylation, relieving inhibitory constraints on autophagy. Fructose metabolism promotes lipid accumulation and contributes to mTORC1 activation. (C) NAD metabolism. Nicotinamide adenine dinucleotide (NAD^+^) depletion, partly driven by poly­(ADP-ribose) polymerase (PARP) activation, reduces sirtuin 1 (SIRT1) activity and promotes acetylation of autophagy-related proteins (ATGs). Reduced nicotinamide adenine dinucleotide phosphate (NADPH) availability contributes to reactive oxygen species (ROS) production and modulation of AMPK-mTORC1 signaling. (D) Lipids. Lipid-derived signals regulate autophagy through organelle-specific mechanisms. Ceramide accumulation at the endoplasmic reticulum (ER) promotes autophagy-related pathways, whereas lysosomal cholesterol depletion negatively influences Rag-dependent mTORC1 signaling. Increased lipid droplets reflect altered lipid storage and metabolic stress than can influence autophagy regulation. Collectively, these pathways converge on ULK and PI3KC3 complexes to regulate autophagy initiation and adaptation to nutrient stress. Solid arrows indicate activation, blunt-ended dotted lines indicate inhibition.

AMPK is a trimeric serine/threonine kinase comprised of a catalytic α-subunit and regulatory β- and γ-subunits. Since multiple isoforms exist for each AMPK subunit, up to 12 heterotrimeric complexes can be assembled, each displaying distinct biochemical properties and tissue-specific expression patterns. AMPK is canonically activated through a mechanism involving adenine nucleotides binding to the γ-subunit, where AMP and ADP compete with ATP.^58^ AMP and ADP binding promotes phosphorylation of a conserved threonine residue (Thr172) within the activation loop of the α-subunit, while also protecting this site from dephosphorylation by phosphatases, thereby increasing net AMPK activity. In addition, Thr172-phosphorylated AMPK can be further allosterically activated upon AMP binding and, to a lesser extent by ADP.^59^ The primary kinase responsible for Thr172-phosphorylation of AMPK is the liver kinase B1 (LKB1). However, this site can also be efficiently phosphorylated by Ca^2+^/calmodulin-dependent protein kinase kinase 2 (CaMKK2) in response to intracellular Ca^2+^ oscillations.^60^

Beyond regulation by upstream kinases, AMPK can be activated by a broad range of synthetic molecules, natural products and fatty acyl-CoAs that bind to a hydrophobic pocket known as the allosteric drug and metabolite (ADaM) site,^61–64^ which is formed at the interface between the kinase domain of the α-subunit and the carbohydrate-binding module of the β-subunit. The ADaM site is further stabilized by phosphorylation of a conserved serine residue in the β1-subunit (Ser108), a site subject to cis-autophosphorylation and also targeted by the autophagy-initiating kinase ULK1.^65^ Interestingly, AMPK complexes lacking phosphorylation at Thr172 can still be substantially activated by ADaM site ligands, provided that Ser108 is phosphorylated.^66,67^ While AMP or ADaM site activators alone have minimal effects on fully dephosphorylated AMPK lacking both α-Thr172 and β1-Ser108 phosphorylation, simultaneous exposure to AMP and an ADaM site compound results in robust synergistic activation of the kinase via a purely allosteric mechanism.^68^

A dual role for AMPK in autophagy regulation has recently been described. On one hand, AMPK-mediated phosphorylation of ULK1 at Ser556 transiently restrains its activation during amino acid starvation, preventing abrupt autophagy induction. On the other hand, AMPK preserves ULK1 and the ATG14-associated PI3KC3 complex from caspase-dependent degradation during energy stress, thereby maintaining the integrity of the autophagic machinery.^69^ This controlled response enables efficient restoration of autophagy and cellular homeostasis once energy stress is resolved.^70^ In addition to these protective effects, upon glucose starvation, AMPK can stimulate autophagy through coordinated regulation of multiple components of the core autophagy machinery. AMPK directly phosphorylates ULK1 at Ser317,^71^ as well as additional sites (Ser467, Ser556, Thr575 and Ser638) that are particularly relevant for mitophagy.^72,73^ In parallel, AMPK increases the binding of Beclin1 to VPS34 and ATG1 by phosphorylating Beclin1 at Thr388.^74^

mTOR is a serine/threonine protein kinase that belongs to the phosphatidylinositol 3-kinase related kinase (PIKK) family and regulates numerous signaling pathways involved in cell growth and metabolism.^75^ It forms the central core of two functionally distinct complexes, mTORC1 and mTORC2. While both complexes share mammalian lethal with SEC13 protein 8 (mLST8), the rapamycin-sensitive mTORC1 contains the scaffold protein Raptor, which regulates both substrate recruitment and subcellular localization,^76,77^ and mTORC2 includes the unrelated scaffold protein Rictor together with the mammalian stress-activated protein kinase-interacting protein 1 (mSIN1).^78,79^ mTORC1 coordinates cellular anabolic metabolism by promoting protein, lipid, and nucleotide synthesis while concurrently inhibiting catabolic processes such as autophagy.^80^ mTORC1 is activated at the lysosomal surface by a small GTPase known as Ras homologue enriched in brain (Rheb) whose activity is in turn regulated by the tuberous sclerosis complex (TSC), composed of TSC1, TSC2, and TBC1D7. TSC2 functions as a GTPase-activating protein (GAP) for Rheb, converting it into inactive GDP-bound state and thereby suppressing mTORC1 activity.^81,82^ Phosphorylation of TSC2 by Akt promotes dissociation of the TSC complex from the lysosomal membrane, preventing inhibition of Rheb and enabling accumulation of active GTP-bound Rheb.^83,84^ In addition to Rheb-dependent regulation, mTORC1 recruitment to the lysosomal surface involves Rag GTPases.^85,86^

mTORC1 is a critical repressor of autophagy. Under nutrient sufficiency conditions, it phosphorylates ATG13 to repress the activity of the ULK1 complex.^71,87^ Additionally, in response to amino acid starvation, mTORC1 directly phosphorylates ATG14 at multiple serine/threonine sites (Ser3, Ser223, Thr233, Ser383, and Ser440) to suppress VPS34 kinase activity.^71,88^ Finally, mTORC1 can modulate autophagy through regulation of lysosomal biogenesis via the transcription factor EB (TFEB),^89^ which controls the expression of numerous lysosomal and autophagy-related genes.^90^ mTORC1 colocalizes with TFEB at the lysosomal membrane, wherein phosphorylates it, promoting TFEB interaction with YWHA, a 14–3–3 family member, thereby sequestering TFEB in the cytoplasm.^91,92^ Thus, by coordinately suppressing TFEB, ULK1 and VPS34-containing complexes, mTORC1 exerts multilayered control over autophagy initiation and lysosomal function. Interestingly, during prolonged starvation, mTORC1 can shift from functioning as an inhibitor of autophagy to supporting the autophagic process. Under conditions of sustained nutrient deprivation, autophagy leads to extensive consumption of lysosomes through autolysosome formation. Recovery of the lysosomal pool depends on mTORC1 reactivation by amino acids generated within autolysosomes, which drives autolysosomal membrane tubulation and the formation of new lysosomes.^93,94^

Nutrients and energy impact on AMPK and mTORC1 to coordinate cellular metabolic homeostasis by regulating autophagy through a reciprocal negative feedback loop. Indeed, under energy-deprived conditions, AMPK negatively regulates mTORC1 by activating TSC2 through phosphorylation of its Thr1227 and Ser1345 residues, but also by direct phosphorylation of the Ser722 and Ser792 residues in Raptor which induces 14–3–3 binding and mTORC1 inactivation; and under nutrient sufficiency conditions, mTORC1 phosphorylates ULK1 at Ser758, thereby disrupting its interaction with AMPK.^71^ This extensive functional crosstalk between AMPK and mTORC1 is underscored by the observation that cells carrying genetic defects that impair AMPK-dependent inhibition of mTORC1 undergo apoptosis under energetic stress.^84,95^ Emerging evidence further indicates that signals generated by distinct nutrients converge at the lysosome to coordinate AMPK and mTORC1 activation, with both kinases in some cases sharing common regulatory interactors.

Therefore, lysosomes act as central platforms that link the degradation of macromolecules to global cellular signaling by integrating metabolic, damage-related, and environmental inputs. The breakdown of biomolecules within the lysosomal lumen generates metabolites such as amino acids, monosaccharides, nucleosides or lipids, which are subsequently transported to the cytosol through specific transporter systems to support cellular metabolism. This transport is functionally coupled to nutrient sensing mechanisms located on the cytosolic lysosomal surface, including mTORC1, where signaling complexes coordinate and regulate growth, catabolism, and stress adaptation.^96^ Therefore, increasingly lysosomes are viewed not only as degradative organelles but also as key integrators of nutrient status and organelle integrity, driving adaptive, tissue-specific physiological responses.^97^

Together, these interconnected mechanisms highlight that autophagy is governed by a multifaceted metabolic sensing network in which nutrients and metabolites act as dynamic regulatory signals rather than passive substrates. Changes in the intracellular availability of amino acids, glucose, lipids and metabolic intermediates are sensed through highly coordinated pathways involving mTORC1, AMPK and lysosomal nutrient sensing. Through these mechanisms, metabolites influence every stage of the autophagic process, from autophagy initiation to lysosomal function and cargo degradation. Autophagy itself reciprocally shapes cellular metabolism by recycling macromolecules and maintaining energetic and biosynthetic homeostasis, establishing a bidirectional crosstalk between metabolic state and autophagic activity. Below we highlight specific mechanisms by which different metabolites impact on autophagy activity.

## Amino Acids

Amino acids are the fundamental units for proteins, the most abundant macromolecules in cells, and their availability is tightly coupled to cellular metabolic demands due to the high energetic cost of protein synthesis. Accordingly, cells continuously monitor both extracellular and intracellular amino acid levels to coordinate anabolic and catabolic processes for organismal growth and development.^98–100^ When intracellular amino acid levels decline, proteins can be mobilized through proteasomal degradation and autophagy, providing recycled amino acids for essential biosynthetic processes and supporting cell survival under nutrient stress. Thus, precise amino acid sensing is essential for balancing protein synthesis, degradation, and broader metabolic homeostasis, as well as for regulating systemic nutrient-related responses.

### mTORC1-Mediated Amino Acid Sensing

While mTORC1 is not a direct amino acid sensor, it is regulated by changes in amino acid availability. The characterization of amino acid-dependent mTORC1 activation started with the identification of the family of Rag GTPases,^86,101,102^ which are members of the Ras superfamily of GTP binding proteins that function as molecular switches linking nutrient availability to cellular growth and metabolism. In mammals, four Rag GTPases have been identified (RagA/B and RagC/D) whose dimerization is essential for the stability of Rag proteins. These heterodimers consisting of RagA or RagB are tethered to the lysosomal membrane by the Ragulator complex, a multiprotein complex consisting of five subunits (LAMTOR1–5) which provides structural support and acts as a scaffold for amino acid-dependent recruitment and activation of mTORC1.^103^ During amino acid sufficiency, RagA/B is GTP-bound and RagC/D is GDP-bound, forming an active heterodimer that interacts with Raptor and promotes mTORC1 recruitment to the lysosomal surface, which leads to its further activation by Rheb, stabilizing its active conformation and enabling it to coordinate a broad range of cellular processes.^104–106^ Two complexes regulate Rag GTPase activity: GATOR1, a heterotrimer that interacts directly with RagA/B acting as a GAP for RagA and RagB;^107^ and GATOR2, a heteropentameric complex that functions as an inhibitor of GATOR1.^108,109^ GATOR1 lysosomal localization is dictated by KICSTOR,^110^ a heterotetrameric complex that directly associates with negatively charged membrane lipids.^111,112^ It has been recently shown that KICSTOR and GATOR1 form a dimeric supercomplex that confines GATOR1 to an orientation toward the low-affinity active GAP mode of Rag GTPases.^113^

In parallel with GATOR-dependent regulation of RagA/B, the folliculin (FLCN)-FNIP complex acts as a GAP for RagC/D, promoting their GDP-bound state in response to amino acid availability.^114^ Under nutrient-deprived conditions, FLCN-FNIP2 associates with the RagA GDP-RagC GTP-Ragulator complex at the lysosomal surface, forming the so-called lysosomal FLCN complex (LFC). This configuration is proposed to restrain FLCN GAP activity.^115^

Structural studies have demonstrated a direct interaction between FLCN and RagC/D,^115,116^ while GDP-bound RagC/D promote the recruitment of MiT/TFE transcription factors, including TFEB and TFE3, to lysosomal mTORC1, thereby facilitating their phosphorylation and subsequently controlling lysosome biogenesis and autophagy.^117,118^

#### Rag GTPase Dependent Mechanisms

Leucine, arginine, methionine and threonine regulate mTORC1 through the Rag GTPase signaling pathway, and some of these amino acids are detected by different sensors.

##### Leucine Sensing

Leucyl-tRNA synthetase (LARS1) functions both as a leucyl-tRNA synthetase and a leucine sensor that regulates mTORC1 directly through Rag GTPases.^119^ Upon leucine binding, LARS1 undergoes conformational changes that promote its interaction with RagD, facilitating the hydrolysis of GTP to GDP and the Rag nucleotide state required for mTORC1 recruitment and activation.^120^ In contrast, Sestrin2 and SAR1B bind to and inhibit the GATOR2 complex under leucine deprivation, favoring GATOR1-dependent suppression of mTORC1.^121,122^ When leucine binds SAR1B and Sestrin2, GATOR2 is released, which results in mTORC1 activation.^122^ SAR1B and Sestrin2 display different leucine-binding affinities and tissue-specific expression patterns, suggesting complementary functions in nutrient sensing and regulation of mTORC1 activity across tissues.^121^

##### Arginine Sensing

CASTOR1 is a cytosolic arginine sensor for mTORC1 that forms homo or heterodimers with its homologue CASTOR2 to regulate mTORC1 signaling through arginine dependent GATOR2 binding.^123,124^ Recent structural evidence has elucidated the molecular basis of this regulation, showing that, under low-arginine conditions, CASTOR1 interacts with GATOR2 through the MIOS subunit. Arginine binding induces conformational changes in CASTOR1 that occlude the MIOS-binding interface, promoting CASTOR1 dissociation from GATOR2 and subsequent mTORC1 activation.^125^

CASTOR1 displays a markedly stronger affinity for arginine than CASTOR2, supporting the idea that these proteins function together to modulate mTORC1 activity across different intracellular arginine levels.^126^ Arginine can also be sensed in the lysosome lumen through the lysosomal amino acid transporter SLC38A9. Arginine binding to the cytosolic domain of SLC38A9 induces a conformational change that enables its interaction with the Ragulator and the stabilization of RagA/B GTP, ultimately facilitating lysosomal recruitment and activation of mTORC1.^127–129^ Additionally, transmembrane 4L six family member 5 (TM4SF5) has been proposed to function as a plasma membrane arginine sensor that interacts with arginine via its extracellular loop. Arginine binding promotes translocation of TM4SF5 from the plasma membrane to lysosomes, where it directly associates with and activates mTORC1.^130^

##### Methionine Sensing

SAMTOR links methionine availability to mTORC1 signaling through dynamic regulation of the GATOR1-KICSTOR complex.^131^ Under methionine-deprived conditions, reduced SAM levels promote SAMTOR association with GATOR1-KICSTOR, resulting in suppression of mTORC1 activity.^131^ Conversely, SAM sufficiency disrupts this interaction and promotes the association of GATOR1 with the protein arginine methyltransferase 1 (PRMT1) and the subsequent methylation of the GATOR1 subunit NPRL2, thereby attenuating GATOR1 activity and facilitating mTORC1 activation.^132^

##### Threonine Sensing

Mitochondrial threonyl-tRNA synthetase 2 (TARS2) has been proposed to function as a threonine sensor. Upon threonine binding, TARS2 interacts with inactive Rag GTPases and promotes GTP loading of RagA, thereby facilitating mTORC1 activation.^133^

#### Rag-GTPase Independent Sensing

In contrast, glutamine and asparagine stimulate lysosomal translocation of mTORC1 via a Rag-GTPase independent mechanism^134,135^ that requires the lysosomal v-ATPase and the ADP-ribosylation factor 1 (ARF1). Although it has been established that Arf1 guanine nucleotide cycling is required for glutamine and asparagine to induce mTORC1 activation and lysosomal localization, the underlying molecular mechanisms remain to be fully elucidated.^135,136^

Moreover, arginine can regulate autophagy through modulation of the TSC-Rheb pathway. By preventing lysosomal accumulation of the TSC complex, arginine promotes Rheb-dependent activation of mTORC1 and suppresses autophagy.^137^

### GCN2-Mediated Amino Acid Sensing

Beyond canonical mTORC1-based amino acid sensing mechanisms, additional nutrient-sensing pathways monitor amino acid availability, among which the General Control Nonderepressible 2 (GCN2) signaling axis plays a central role. Amino acid depletion leads to the accumulation of uncharged tRNAs which bind to GCN2 causing a conformational change and autophosphorylation that allows GCN2 to phosphorylate and inhibit its downstream target, the eukaryotic translation initiator factor (eIF2α).^138^ This phosphorylation restricts the efficient initiation of translation for most mRNAs and selectively enhances translation of stress-responsive mRNAs, including ATF4, which initiates a transcriptional cascade that regulates genes involved in apoptosis, autophagy, and amino acid metabolism.^139^ This adaptive response upregulates specific aminoacyl-tRNA synthetases and amino acid transporters, thereby enhancing amino acid uptake to sustain protein synthesis under nutrient-deficient conditions.

Additionally, a recent study has shown that depletion of glutamine and asparagine is sensed through GCN2, which initiates Rag-dependent and Rag-independent mechanisms to inhibit mTORC1, pointing to an additional role of GCN2 in the control of mTORC1.^140^ Independently of its function as an eIF2 kinase, GCN2 inhibits mTORC1 through the Rag GTPases; but also, through ATF4 induction, upregulates REDD1, an inhibitor of mTORC1 through the TSC complex;^141,142^ and Sestrin2, which together cause additional mTORC1 inhibition.^140^ Concurrently, GCN2 phosphorylates and increases FBXO22 activity, leading to mTOR ubiquitination at Lys2066 and suppression of mTORC1 signaling.^143^ This additional nutrient-stress sensing mechanism supports metabolic adaptation under amino acid.

## Glucose and Glycolysis Intermediates

### Glucose Availability

Glucose is the primary carbon and energy source for most mammalian cells, and fluctuations in glucose availability substantially influence autophagic flux through multiple interconnected mechanisms. Historically, glucose deprivation was understood primarily as a trigger of autophagy through changes in cellular energy charge. These early studies, primarily performed in yeast, demonstrated that glucose withdrawal slows glycolytic flux, increasing the AMP/ATP ratio and activating the central energy-sensing kinase AMPK.^144–146^ AMPK activation was subsequently shown to promote autophagy through direct phosphorylation of ULK1 (particularly Ser555), inhibition of mTORC1 activity, and broader reprogramming toward a catabolic cellular state.^71,72^ These findings established a foundational model in which glucose regulates autophagy indirectly through its contribution to cellular energy status, forming the basis of many studies identifying correlational evidence between glucose withdrawal, increased ULK1 phosphorylation and heightened AMPK activity.^147^

More recent work has expanded this view, however, demonstrating that glucose availability can directly regulate autophagy through mechanisms that are not solely dependent on changes in adenine nucleotide ratios. For instance, glucose deprivation has been shown to promote ULK1-dependent phosphorylation of the lipid kinase PIKfyve on Ser1548, increasing the synthesis of phosphatidylinositol-5-phosphate (PI(5)­P) and driving the formation of PI(5)­P-enriched autophagosomal membranes.^148^ This pathway represents a noncanonical mechanism of autophagy induction that operates independently of the classical VPS34-PI3P axis,^149^ suggesting that glucose-derived signals can directly influence membrane dynamics associated with autophagosome biogenesis.

In addition to these downstream effects, glucose metabolism can also be directly sensed by the upstream autophagy-related nutrient sensing machinery. Mechanistic studies have shown that the glycolysis enzyme aldolase functions as a sensor of the glycolytic intermediate fructose-1,6-bisphosphate (FBP), such that reductions in FBP during glucose withdrawal promote the assembly of a lysosomal AMPK activation complex containing LKB1 and the v-ATPase.^150^ This pathway enables activation of a lysosome-localized pool of AMPK, facilitating rapid and spatially restricted suppression of mTORC1 signaling prior to measurable changes in cellular adenylate energy charge.^150,151^ These findings support a model in which glucose availability is sensed not only through ATP depletion, but through metabolite-dependent signaling platforms that couple glycolytic flux directly to autophagy regulation.

It must be mentioned, however, that the relationship between glucose availability, AMPK activity and autophagy regulation continues to be challenged and revised. Emerging evidence indicates that under energy stress, direct or glucose withdrawal-induced AMPK activity can suppress both bulk and selective autophagy initiation by inhibitory phosphorylation or 14–3–3-mediated sequestration of ULK1,^152,153^ even during conditions of amino acid withdrawal. This inhibitory influence, primarily mediated by phosphorylation of ULK1 at Ser556/694,^153^ has been proposed as an energy-preserving function to limit ATP consumption when energy is critically low, thus positioning AMPK as a context-dependent regulator of autophagy in response to fluctuating glucose availability.^154^

Together, these findings demonstrate that glucose and related metabolites regulate autophagy through a multilayered nutrient-sensing network that integrates cellular energy charge, metabolite availability and spatially organized signaling platforms. As such, carbohydrate metabolism determines the magnitude and directionality of autophagy responses via mechanisms expanded upon further below.

### Glycolytic Intermediates

#### UDP-GlcNAc

A range of metabolic byproducts of glucose metabolism has been reported to regulate autophagy, integrating signals from multiple nutrient inputs that similarly converge on the initiation machinery, typically the ULK1 kinase complex. An example of this comes from the hexosamine biosynthetic pathway (HBP), where a proportion of intracellular glucose is channelled away from glycolysis into anabolic processing.^155^ In the HBP, fructose-6-phosphate is converted to UDP-N-acetylglucosamine (UDP-GlcNAc), a process that requires not only glucose, but also glutamine, acetyl-CoA and UTP, thereby coupling carbohydrate, amino acid, lipid and nucleotide metabolism into a multinutrient sensing pathway.^155^ Through this pathway, fluctuations in nutrient availability are translated into changes in protein O-GlcNAcylation, an abundant, dynamic post-translational modification that broadly reflects the cellular metabolic state.^155^

Growing evidence indicates that O-GlcNAcylation behaves as a nutrient-responsive regulator of multiple components of the autophagy machinery.^156^ For example, during initiation, it has been shown that ULK1 modifications during glucose starvation extend beyond classical phospho-regulatory elements, and that O-GlcNAcylation of ULK1 at Thr754 correlates with autophagosome formation, in direct opposition to the inhibitory phosphorylation mediated by mTORC1 on Ser757.^157^ This mechanism results in enhanced ULK1-ATG14 interactions, previously shown to be essential for optimal autophagy initiation.^152^ However, like glucose metabolism, the effect of O-GlcNAcylation on autophagy appears to be context dependent and strongly influenced by the extent of protein modification and nutrient availability. It has been shown that in the brain of Parkinson’s disease patients, global O-GlcNAcylation is increased, and experimental elevation of O-GlcNAc levels suppresses autophagic flux.^158^ Mechanistically, increased O-GlcNAcylation was associated with increased AKT-dependent mTORC1 activation, an effect that was reversible by rapamycin treatment.^158^ These findings are consistent with work examining the later stages of autophagy, showing that O-GlcNAcylation of the SNARE protein SNAP-29 in cancer cells impairs autophagosome-lysosome fusion, thereby blocking autophagic flux.^159^ As O-GlcNAcylation is generally increased during nutrient-replete conditions,^160^ these studies support the concept that O-GlcNAcylation integrates multiple nutrient signals to dynamically regulate autophagy depending on metabolic state and duration of nutrient exposure.

#### Acetyl-CoA

In a manner similar to the HBP, acetyl-CoA links nutrient availability to autophagy, by coupling carbon processing to post-translational protein acetylation modifications. Accordingly, cytosolic acetyl-CoA sits at the intersection of glucose, lipid and amino acid metabolism, broadly reflecting general nutrient sufficiency, although up to 90% of this is generated from glucose under normoxic conditions.^161^ As the sole donor of acetyl groups for acetyltransferase reactions, acetyl-CoA directly influences protein acetylation. Early mechanistic work showed that nutrient withdrawal rapidly lowers acetyl-CoA, global protein acetylation levels and induces autophagy, an effect that can be reversed by restoring acetyl-CoA levels through inhibition of pyruvate dehydrogenase kinase.^162^ A crucial mediator of autophagy suppression under conditions of acetyl-CoA sufficiency is the acetyltransferase EP300,^162^ which has been shown to directly acetylate components of the autophagy machinery, including Beclin1, PIK3C3 and LC3, to inhibit autophagosome formation.^163–167^ Recent work has extended this principle further by showing that reductions in cytosolic acetyl-CoA following short-term nutrient restriction can also act as a trigger for NLRX1-dependent mitophagy,^168^ suggesting that acetyl-CoA availability, reflective of nutrient abundance, may influence not only the magnitude of bulk autophagy, but also aspects of cargo selectivity.

#### NADPH and Redox Signaling

In addition to UDP-GlcNAc and acetyl-CoA, glucose processing also regulates autophagy through the generation of NADPH, a key redox cofactor produced primarily through the oxidative pentose phosphate pathway (PPP).^169^ NADPH plays a critical role in maintaining cellular redox balance by supporting antioxidant systems, including glutathione and thioredoxin, which buffer reactive oxygen species (ROS) and prevent oxidative damage.^170^ At the same time, NADPH also serves as a substrate for NADPH oxidases (NOX), enabling controlled ROS generation as a signaling intermediate.^171^

Through these dual roles, NADPH links glucose metabolism to autophagy via redox-dependent signaling mechanisms. ROS have been widely implicated in the regulation of autophagy, functioning not only as damaging byproducts of metabolism, but also as signaling molecules that modulate autophagy-related proteins.^172–174^ One key mechanism involves the reversible oxidation of cysteine residues within the core autophagy machinery, such as ATG4, which regulates LC3 processing, and the oligomerization of adaptor proteins including p62 and NDP52, thereby influencing both autophagosome formation and cargo selection in specialized processes such as mitophagy.^175–177^ Such reversible oxidation reactions are currently thought to render components of the autophagy machinery sensitive to changes in the intracellular redox environment,^178,179^ promoting specialized processes such as PINK1/Parkin-dependent mitophagy as a cellular response to oxidative stress^177^

Accordingly, fluctuations in NADPH availability can influence autophagy by affecting the balance between ROS production and antioxidant buffering capacity. Nutrient deprivation can disrupt cellular redox homeostasis by limiting the availability of reducing equivalents such as NADPH and glutathione, thus promoting a more oxidative intracellular environment.^173^ Under conditions of high NADPH availability, enhanced antioxidant capacity may suppress ROS-related signaling, whereas reduced NADPH levels could promote oxidative stress and autophagy activation.^180,181^ In this way, the PPP functions as a nutrient-sensitive regulator of autophagy, translating central carbon metabolism into redox signaling events that promote or suppress autophagy activity and selectivity.

### Other Sugars and Carbon Sources

While much of the mechanistic understanding of carbon-dependent autophagy regulation has been derived from studies of glucose metabolism, other sugars can also influence autophagy by altering metabolic flux and nutrient-sensing pathways. Unlike glucose, which is directly linked to canonical nutrient-sensing networks, alternative sugars typically regulate autophagy indirectly by reshaping metabolic activity, energy production and redox balance. As such, these sugars provide useful models for understanding how changes in carbon source availability and utilization influence autophagy through broader metabolic reprogramming, rather than dedicated sensing mechanisms.

#### Fructose

Fructose represents a major component of modern caloric intake that can serve as an alternative carbon source for mammalian metabolism. In contrast to glucose, fructose metabolism largely occurs in hepatocytes and bypasses key regulatory steps of glycolysis through phosphorylation by fructokinase and subsequent cleavage by aldolase B, rapidly generating triose phosphates that feed both lipid synthesis and glycolysis.^182^ This unregulated entry into central carbon metabolism enables fructose to drive sustained anabolic flux, even under conditions where glycolytic control would otherwise limit carbon utilization.^183^

Consistent with this alternative metabolic processing, a number of studies report that chronic fructose exposure suppresses hepatic autophagy and promotes lipid accumulation. High-fructose feeding in laboratory animals activates hepatic mTORC1 signaling, which inhibits the ULK1 complex and downstream autophagy initiation, contributing to hepatic steatosis and metabolic dysfunction.^184,185^ Fructose-driven lipogenesis and associated nutrient signaling reinforce an anabolic cellular state that suppresses autophagy initiation,^186^ contributing to numerous downstream events including inhibition of autophagic cell death and enhanced pancreatic cancer cell survival.^187^ Such studies highlight a physiological role for fructose metabolism in redirecting carbon flux toward growth and storage pathways, in part through activation of kinases that have evolved to signal nutrient excess and suppress autophagy.

#### Galactose

In contrast to glucose, galactose metabolism yields no net ATP from aerobic glycolysis, forcing cells to rely predominantly on mitochondrial oxidative phosphorylation to maintain energy homeostasis.^188^ This metabolic switch is widely used as an experimental tool to reveal mitochondrial dysfunction commonly masked under glycolytic conditions, and, given the close links between mitochondria and autophagy, this shift toward respiratory metabolism can alter mitochondrial quality-control pathways such as mitophagy. Indeed, many glucose-addicted immortalised cell models only exhibit detectable basal mitophagy activity upon galactose culture, reflecting increased dependence on mitochondrial function and associated ROS generation.^189,190^

Accordingly, this metabolic response to prolonged galactose reliance appears to be a stress response to mitochondria-associated ROS, and has been shown to mediate premature senescence-like phenotypes in epithelial models, associated with downstream alterations in autophagy.^191^ In this context, galactose metabolism may influence autophagy indirectly by promoting mitochondrial stress and redox imbalance, thereby engaging quality-control pathways rather than classical nutrient-sensing mechanisms. This metabolic input, although not strictly integrated with the nutrient-sensing machinery like glucose, illustrates how alternative carbon sources can modulate autophagy indirectly through metabolic reprogramming and mitochondrial stress, rather than direct nutrient signaling.

## NAD

In addition to NADPH-dependent antioxidant systems, cellular metabolism is critically regulated by the redox pairing of NAD^+^ and NADH (collectively NAD), which function as central cofactors linking nutrient availability to metabolic activity. Although not traditionally classified as a nutrient, intracellular NAD levels reflect metabolic state and act as key determinants of cellular bioenergetic capacity and similarly regulate autophagy primarily through indirect mechanisms.^5,192^ In this context, NAD ties together signals derived from glucose metabolism and mitochondrial function, positioning it as a central mediator of nutrient-dependent autophagy regulation.

Beyond its role in redox metabolism, NAD^+^ also serves as a direct substrate for a class of enzymes collectively termed NAD^+^-consuming enzymes (NADases). Such enzymes include the sirtuin (SIRT) deacetylases, Poly-ADP-ribose-polymerase (PARP), CD38, CD157 and SARM1 families, all of which play roles in cellular repair and protein modifications and have been implicated in the control of autophagy activity. Among these, SIRT1 represents the most characterized link between NAD availability and autophagy regulation.^193^

SIRTs are NAD^+^-dependent deacetylases, and early work demonstrated that knockout of *Sirt1* in mouse embryonic fibroblasts (MEFs) enhanced ATG5/7/8 acetylation and impaired p62 clearance, a marker commonly indicative of impaired autophagy.^194^ Further work has since built on this mechanism, showing that Sirt1 downregulation in mice is associated with both ATG5/7 acetylation and impaired ATG5-ATG12 conjugation, implying a positive role of Sirt1 activity in the elongation step of autophagosome synthesis.^195^ These findings established a model in which NAD^+^ availability promotes autophagy through SIRT1-dependent deacetylation of the core autophagy machinery.

Building on this work, more recent studies suggest that SIRT1 regulates autophagy at multiple levels beyond acetylation-dependent ATG conjugation, positioning it as a broader metabolic sensor that couples NAD^+^ availability with autophagic flux. For example, SIRT1 has been shown to act upstream of ATG kinetics by shifting nutrient sensing signaling toward a pro-autophagy AMPK “on” mTOR “off” catabolic phenotype, particularly in cancer cell lines.^196,197^ Beyond initiation, SIRT1 has also been shown to function in the promotion of autophagic flux, by enhancing autophagosome-lysosome fusion and substrate clearance by increasing Rab7 abundance, under conditions of oxidative stress. Such changes in autophagy-related protein expression may arise from the well-documented transcriptional control driven by SIRT1 activity. These effects are consistent with the well-established transcriptional role of SIRT1, whereby deacetylation of FOXO transcription factors enhances expression of genes involved in autophagy and lysosomal function, including LAMP2.^198,199^ Together, these findings position SIRT1 as a key mediator of both the initiation and execution of autophagy, linking NAD^+^ availability to nutrient-related signaling. Indeed, boosting intracellular NAD pools has been shown to enhance autophagy and restore cellular function in multiple disease models.^200–202^

Poly­(ADP-ribose) polymerases (PARPs) regulate numerous cellular processes by catalyzing the transfer of ADP-ribose moieties from NAD^+^ onto target proteins. In contrast to sirtuins, which generally promote autophagy through NAD^+^-dependent protein deacetylation, PARPs consume NAD^+^ during DNA damage and stress responses, thereby linking genotoxic stress to autophagy through distinct mechanisms. Activation of PARP1, the most abundant and catalytically active PARP family member, can rapidly deplete intracellular NAD^+^ and ATP pools, an effect that can be rescued by NAD precursor supplementation.^202,203^ This depletion of NAD^+^ indirectly limits SIRT1 activity while simultaneously inducing energetic stress,^204^ thereby altering autophagy through both signaling and metabolic pathways. This PARP1-driven metabolic shift has been shown to influence key nutrient-sensing pathways, including AMPK activation and mTORC1 suppression, particularly in cancer models.^205^ Accordingly, PARP activity has been proposed as a mechanism through which changes in NAD availability integrate stress responses with autophagy regulation.

Similar to SIRT1, the role of PARP1 in autophagy appears to be highly context dependent. Early studies demonstrated that DNA damage-induced PARP1 activation promotes cytoprotective autophagy in MEFs,^206^ while depletion of PARP1 in oxidative stress models impaired autophagy and increased mitochondrial function.^207^ However, more recent work indicated that excessive of prolonged PARP1 activation can suppress autophagy, largely through depletion of NAD^+^ and disruption of downstream metabolic processes. For example, in ARPE-19 cells exposed to oxidative stress, PARP1-mediated NAD^+^ depletion impaired autolysosome formation, an effect that was rescued by NAD precursor supplementation or PARP inhibition by olaparib.^203^ Notably, this rescue was absent in SIRT1-deficient cells, suggesting that PARP1-mediated suppression of autophagy occurs, at least partially, through limiting NAD^+^ availability for SIRT1-dependent pathways.

Together, these findings support the view that PARP1 functions as a stress-responsive regulator of autophagy, capable of both promoting and suppressing autophagy depending on the extent of NAD^+^ consumption and cellular energy status. This is consistent with a broader model of PARP1 in determining cell fate, where moderate activation supports DNA repair and survival, whereas excessive activation leads to NAD^+^ depletion and a form of programmed cell death known as parthanatos,^208^ which has been implicated in Parkinson’s disease pathology.^209^

In addition to its role as a substrate for NAD-consuming enzymes, emerging evidence suggests that the NAD^+^/NADH redox ratio itself can act as a direct regulator of autophagy. For instance, an elevated NADH/NAD^+^ ratio, indicative of reductive stress, has been shown to promote autophagy induction through activation of AMPK and phosphorylation of ULK1 at Ser555.^210^ Notably, this effect was observed across multiple experimental approaches to increase NADH levels, including direct NADH supplementation, and was dependent on both LKB1 and CAMKK2 signaling pathways linked to AMPK activation. Consistent with this, restoration of NAD^+^ levels through nicotinamide supplementation has been shown to rescue autophagy defects in metabolic disease models by suppressing mTORC1 signaling via the IGF-1/AKT pathway.^211^ Importantly, these effects occurred independently of SIRT1, as no changes in ATG protein acetylation were observed, indicating that NAD can regulate autophagy through mechanisms distinct from NADase-dependent protein deacetylation.

Together, these findings support the concept that NAD functions as a nutrient-responsive signaling molecule, capable of directly engaging canonical autophagy regulatory pathways, similar to other nutrient metabolites like amino acids and adenylates. Additionally, by alleviating cellular stress, autophagy has itself been shown to maintain intracellular NAD levels,^202,212,213^ suggesting a bidirectional relationship in which NAD regulates autophagy and nutrient-responsive signaling, while autophagic activity contributes to healthy NAD homeostasis. In this model, NAD and autophagy are interconnected, with NAD integrating metabolic flux, redox balance and energy status to influence autophagic activity, and autophagy reciprocally supporting metabolism through the maintenance of NAD availability. These enzyme-dependent and enzyme-independent mechanisms provide an additional layer of regulation linking cellular energetic status to autophagy, beyond classical adenylate signaling associated with glucose deprivation.

## Lipids

Lipids comprise a diverse group of hydrophobic nutrients that are essential for energy storage, membrane biosynthesis, and cellular signaling. Although the mechanisms underlying cellular lipid sensing remain poorly defined, these metabolites have increasingly been recognized as key regulators of autophagy, acting at multiple stages of the pathway, from autophagosome biogenesis to cargo degradation.

Autophagy is tightly linked to lipid metabolism, particularly triglyceride turnover, as it is dynamically regulated by cellular nutrient availability. Lipids are stored as triacylglycerols, cholesterol esters, and retinyl esters within lipid droplets (LDs), intracellular organelles enclosed by a phospholipid monolayer whose surface is coated by perilipin proteins, which are key regulators of LDs metabolism. When glucose becomes limited, cells undergo a metabolic switch from carbohydrate utilization to lipid-based energy production and the stored lipids can be mobilized through lipolysis and lipophagy.^214^ This leads to the release of free fatty acids (FFAs) that are subsequently transported to mitochondria for β-oxidation.^215,216^ Conversely, autophagy has also been implicated in the regulation of triglyceride biosynthesis. Indeed, LD-associated protein Perilipin-2 protects LDs from autophagic degradation and modulates lipid metabolism in liver, as its depletion enhances autophagy while reducing lipogenesis and triglyceride synthesis.^217,218^

Thus, despite their role as essential nutrients, lipids can also act as autophagy inducers, likely serving as a mechanism to prevent lipotoxicity^219^ and LD biogenesis might represent a universal cellular response during periods of enhanced autophagic activity, providing a lipid buffering system that mitigates lipotoxic damage.^220^ How lipids contribute to lipotoxicity-induced autophagy remain unclear. In this context, FFAs such as palmitic acid (PA), a potent lipotoxic mediator, have been reported to exert dual effects on autophagy, either inducing or inhibiting the process depending on concentration and duration of exposure. Its pro-autophagic activity has been shown to occur independently of mTORC1 and involves activation of PKC and JNK signaling pathways in hepatocytes,^221,222^ thus leading to Beclin-1 dissociation from Bcl-2 and autophagy initiation.^223^ In contrast, prolonged PA exposure disrupts autophagic flux by impairing autophagosome-lysosome fusion.^224^ More recently, PA has been shown to inhibit hepatic autophagy through suppression of the immune sensor DDX58/Rig-1 and activation of the STING-mTORC1 axis, mechanisms that may contribute to autophagy impairment in advanced Non-Alcoholic Fatty Liver Disease.^225,226^

Through a different mechanism, myristic acid upregulates Beclin-1 and Atg7 expression and enhances autophagic flux in mouse cardiomyocytes. This pro-autophagic effect is thought to be mediated through the generation of C_14_-ceramide and the upregulation of ceramide synthase 5.^227^ Myristic acid is not lipotoxic, however it can potentiate PA lipotoxicity^228^

Although fatty acids are major regulators of autophagy, multiple other lipid species also modulate autophagic activity, often exerting diverse and sometimes opposing effects depending on the lipid class, cellular context, and duration of exposure.

### Lipid Signaling Molecules

#### Cholesterol

Cholesterol biosynthesis and autophagy appear to be inversely related, such that inhibition of cholesterol synthesis is associated with autophagy induction. This is supported by evidence showing that statin activates autophagy in human blood cancer cells through mTORC1 pathway inhibition and that cholesterol depletion alone, induced by methyl-β-cyclodextrin, is sufficient to trigger a similar autophagic response.^229^ In this line, deficiency of transmembrane 7 superfamily member 2 (TM7SF2), a positive regulator of cholesterol biosynthesis, suppresses autophagy.^230^ Extending these observations, it has been shown that cholesterol depletion regulates autophagy through a dual mechanism involving transcriptional activation of autophagy-related genes and impairment of autophagic flux. Notably, this latter effect depends on ROS accumulation and JNK activation.^231^ Within this framework, inhibition of cholesterol efflux from late endosomes caused by mutations in the intracellular cholesterol transporter NPC1, leads to the accumulation of autophagosomes and defective autophagy.^232^ Overall, these findings underscore its complex regulatory role in cellular homeostasis.^231^

Indeed, cholesterol can also regulate autophagy by acting as a lysosomal nutrient signal upstream of mTORC1. This response is mediated by LYCHOS (GPR155), a lysosomal transmembrane cholesterol sensor that links cholesterol availability to mTORC1 activation.^233,234^ Structural studies have further revealed that LYCHOS combines transporter- and GPCR-like domains closely associated with the region involved in cholesterol-dependent mTORC1 signaling, suggesting a functional coupling between metabolite transport/sensing and downstream signaling.^235^ Under cholesterol-replete conditions, LYCHOS interacts with GATOR1 through a conserved cytosol-facing region, thereby limiting its inhibitory control over the Rag GTPases. This promotes Rag-dependent recruitment of mTORC1 to the lysosomal surface, providing a direct molecular link between lysosomal cholesterol sensing and mTORC1 signaling.^233^

#### Phospholipids

While PE, the second-most abundant phospholipid in mammalian membranes,^236^ primarily supports autophagosome membrane dynamics through LC3 lipidation,^237^ other phospholipids, particularly phosphoinositides constitute a distinct class of signaling molecules in the regulation of autophagy initiation.

Upon nutrient availability, the class I phosphoinositide 3-kinase (PI3K) phosphorylates phosphatidylinositol 4,5-bisphosphate (PIP_2_) to generate phosphatidylinositol 3,4,5-trisphosphate (PIP_3_).^238^ PIP_3_ activates PDK1, which in turn phosphorylates Akt,^239^ leading to mTORC1 activation and suppression of autophagy.^240–242^ In contrast, under nutrient-deprived conditions, phosphatase and tensin homologue (PTEN) dephosphorylates PIP_3_, reversing this signaling cascade, resulting in mTORC1 inhibition and autophagy induction.^243^ On the other hand, PI3P, generated either through phosphorylation of phosphatidylinositol (PI) by class III PI3K or through dephosphorylation of PIP_3_ by phosphatases, exemplifies the spatial regulation of autophagy-related signaling. Spatially restricted production of PI3P in subdomains of the ER is critical for the initial steps of autophagosome biogenesis and contributes to autophagosomal membrane formation, expansion and sealing;^244^ and facilitates selective cargo sequestration through its interaction with autophagy-linked FYVE protein (Alfy).^245^

#### Sphingolipids

Sphingolipids are abundant components of eukaryotic plasma membranes that participate in a broad range of biological processes, including autophagy.^246,247^ Among them, ceramide has emerged as an important inducer of autophagy through multiple interconnected pathways involving both mTORC1- and AMPK-dependent mechanisms. On one hand, ceramide activates phosphoprotein phosphatase 2A (PP2A), which inhibits Akt signaling within the class I PI3K pathway, thereby suppressing mTORC1 activity.^248^ On the other hand, ceramide can promote AMPK activation^249^ through downregulation of nutrient and amino acid transporters, leading to metabolic stress and ultimately, autophagy induction.^247,250^ Simultaneously, ceramide facilitates dissociation of the Beclin1-Bcl-2 complex, partly through JNK1-dependent mechanisms,^251^ enabling formation of the Beclin1-VPS34-VPS15 complex required for autophagosome biogenesis.^247^ These findings highlight ceramide as a central lipid mediator linking metabolic stress to autophagy induction. Nonetheless, ceramide also represents a key lipid involved in lipid transfer during the elongation of autophagosome biogenesis^252–254^ by promoting vesicle tethering and intervesicular lipid mixing mediated by GABARAP and GABARAPL1.^255^

Derived from ceramide metabolism through the action of sphingosine kinases 1 and 2 (SK1 and SK2), sphingosine-1-phosphate (S1P) promotes autophagy and, unlike ceramide, confers protection against starvation-induced apoptosis.^256^ Mechanistically, S1P-mediated autophagy suppresses mTORC1 activation independently of the class I PI3K signaling or Akt, while enhancing Beclin-1 expression, ultimately promoting cell survival. Conversely, loss or inhibition of SK1 impairs cellular adaptation to stress and can trigger autophagic cell death.^256^ In line with this, accumulation of S1P following inhibition of its degradation induces ER stress, activation of the unfolded protein response, and mTORC1-independent autophagy.^257^

Concomitantly with the broader regulation of autophagy by ceramide metabolism, dhCeramide (dhCer) has been reported to stimulate autophagy in gastric and colon cancers,^258^ and its intracellular accumulation enhances cytotoxicity in cancer cells^259,260^ suggesting that it may function as a regulatory signal determining cell fate.^261^ Likewise, gangliosides^262^ have been reported to induce autophagy-associated cell death in isolated astrocytes and astrocytoma cells.^263^

Extending the role of ceramide-derived sphingolipids, sphingomyelin has been identified as a key regulator of ATG9A trafficking and the maturation of early autophagic membranes.^264^ Its accumulation, which occurs in Niemann-Pick diseases type A and B, leads to defective maturation and impaired closure of autophagic structures.^265^

## Autophagy under Severe Energy Limitation

Recent research has begun to highlight increasingly more complex relationship between energy stress, nutrient withdrawal and autophagy regulation, highly dependent on the degree of stress and cell type.^137,266^ For stressed cells, autophagy is a significant energy investment, requiring ATP at almost all stages of vesicle formation, trafficking and fusion.^267^ As highlighted in numerous recent studies, autophagy activation appears to require a minimum threshold of intracellular ATP for both its initiation and completion, challenging the classical view that energy depletion uniformly acts as a signal promoting autophagy.^268^

Building on this concept, a number of experimental models now demonstrate that severe energetic collapse can actively suppress autophagy initiation, even under nutrient conditions that would normally promote autophagy. For example, prolonged inhibition of mitochondrial oxidative phosphorylation (OXPHOS) by rotenone-induced pharmacological blockade of the electron transport chain in SH-SY5Y cells has been shown to result in suppressed autophagosome formation and severe ATP depletion.^269,270^ Similar work in acute myeloid leukemia cells has shown that multiple independent methods to inhibit OXPHOS, including complex III inhibition by antimycin A or complex I inhibition by IACS-010759 or metformin similarly impaired autophagic flux,^271^ implying a key role of mitochondrial ATP generation in the initiation of autophagy.

Genetic models further support this principle and illustrate how mitochondrial function is tied to nutrient-dependent autophagy regulation. In yeast, cells lacking mitochondrial DNA (Rho^0^), and therefore incapable of mitochondrial OXPHOS,^272^ display impaired autophagic flux during amino acid starvation when cultured in galactose media, accompanied by defective induction of core autophagy machinery such as ATG8.^273^ Mechanistically, respiratory deficiency elevates protein kinase A (PKA) signaling, impairing recruitment of the Atg1-Atg13 complex and suppressing autophagosome formation despite traditional starvation signals.^273^ Consistent with this, mammalian Rho^0^ cells have been shown to similarly display defective autophagic flux, even in the presence of pharmacological mTORC1 inhibition by rapamycin.^274^ Together, these works further support a role of mitochondrial respiration in promoting autophagy induction, by indicating that inhibition of mitochondrial respiration can inhibit autophagy, even in the presence of typical autophagy-inducing nutrient signals such as amino acid withdrawal and mTORC1 activation.

Taken together, these observations suggest that autophagy is regulated within a defined energetic window, in which nutrient-sensing pathways are constrained by cellular energy availability. Under moderate energy stress, canonical signaling through AMPK may promote autophagy initiation via ULK1 activation and mTORC1 inhibition.^71^ However, as ATP levels decline further, this relationship may become uncoupled and autophagy inhibited despite a pro-autophagy nutrient signaling profile. As previously mentioned, recent studies indicate that AMPK may contribute to this response by suppressing autophagy initiation under conditions of severe energy stress while preserving components of the autophagy machinery, thereby limiting ATP expenditure and maintaining catabolic potential during acute energy crisis.^152,153,275^ Collectively, these findings suggest that autophagy is sensitive not only to nutrient availability, but also to the degree of cellular energy depletion, requiring sufficient bioenergetic cellular capacity for both its initiation and completion.

## Metabolite Control of Autophagy during Aging

Aging is characterized by progressive metabolic alterations affecting energy production, glucose regulation, lipid metabolism, and protein synthesis, ultimately resulting in a metabolic drift that impacts energy homeostasis, nutrient sensing, and stress adaptation.^276,277^ These changes profoundly affect autophagy, a metabolically sensitive process whose efficiency or capacity generally declines with age, largely due to changes in metabolite availability and organelle function. This decrease in autophagy establishes a mechanistic link between impaired nutrient sensing and defective cellular quality control. In this context, the crosstalk between autophagy and cellular metabolism is essential for maintaining cellular homeostasis and physiological integrity, while protecting against age-associated pathologies. Accordingly, compromised autophagy has been recognized as one of the hallmarks of aging.^276^

A decline in autophagic function has been frequently associated with aging and age-related disorders.^1,278^ In parallel, reductions in NAD levels have been reported in specific tissues during aging, despite recent inconsistent findings in peripheral blood or broader cohorts.^279^ Consistent with tissue-specific alterations, magnetic resonance (MR)-based in vivo NAD assays have revealed progressive reductions in NAD^+^, NAD^+^/NADH ratio, and total NADH levels in the brains of healthy individuals during aging.^280^ Moreover, disrupted NAD^+^ homeostasis may exacerbate the energetic deficits observed during the presymptomatic stages of neurodegenerative diseases.^281^ Decreased NAD^+^ levels are linked to alterations in mitochondrial function, elevated ROS production, and reduced oxidative metabolism.^282,283^ This is followed by a metabolic shift from oxidative phosphorylation to glycolysis, stabilization of hypoxia-inducible factor 1α (HIF-1α), and reduced activity of mitochondrial respiratory complexes, all of which have been associated with metabolic syndrome, obesity, type 2 diabetes, tumor progression, and neurodegeneration.^284^ Therefore, NAD^+^ and autophagy establish a bidirectional feedback loop that may contribute to the regulation of longevity and the development of age-related diseases.^5^

Concurrently, aging is associated with progressive changes in lipid metabolism that are coupled to mitochondrial dysfunction and contribute to lipotoxic stress, including dysregulated cholesterol homeostasis, reduced lipid oxidation, ectopic lipid accumulation and impaired lipid turnover. These alterations are tightly related to dysregulated nutrient-sensing mechanisms and promote lipid deposition in metabolic tissues such as the liver and muscle, contributing to the development of atherosclerosis and other age-related diseases.^285,286^

Collectively, these metabolic alterations converge on the autophagy machinery, leading to a progressive decline in autophagic flux which further aggravates mitochondrial dysfunction and lipid accumulation, thereby establishing a self-reinforcing feedback loop that amplifies metabolic deterioration and loss of cellular homeostasis during aging.
